# An analysis of genetically regulated gene expression across multiple tissues implicates novel gene candidates in Alzheimer’s disease

**DOI:** 10.1186/s13195-020-00611-8

**Published:** 2020-04-16

**Authors:** Zachary F. Gerring, Michelle K. Lupton, Daniel Edey, Eric R. Gamazon, Eske M. Derks

**Affiliations:** 1 Translational Neurogenomics Laboratory, QIMR Berghofer Medical Research Institute, 300 Herston Road, Herston, Brisbane, QLD 4006 Australia; 2 Genetic Epidemiology Laboratory, QIMR Berghofer Medical Research Institute, 300 Herston Road, Herston, Brisbane, QLD 4006 Australia; 3 Division of Genetic Medicine, Department of Medicine, Vanderbilt University, 1211 21st Ave S, Nashville, TN 37212 USA

**Keywords:** Alzheimer’s disease, Gene expression, Genome-wide association study, Genetics, Genetic epidemiology, Computational biology

## Abstract

**Introduction:**

Genome-wide association studies (GWAS) have successfully identified multiple independent genetic loci that harbour variants associated with Alzheimer’s disease, but the exact causal genes and biological pathways are largely unknown.

**Methods:**

To prioritise likely causal genes associated with Alzheimer’s disease, we used S-PrediXcan to integrate expression quantitative trait loci (eQTL) from the Genotype-Tissue Expression (GTEx) study and CommonMind Consortium (CMC) with Alzheimer’s disease GWAS summary statistics. We meta-analysed the GTEx results using S-MultiXcan, prioritised disease-implicated loci using a computational fine-mapping approach, and performed a biological pathway analysis on the gene-based results.

**Results:**

We identified 126 tissue-specific gene-based associations across 48 GTEx tissues, targeting 50 unique genes. Meta-analysis of the tissue-specific associations identified 73 genes whose expression was associated with Alzheimer’s disease. Additional analyses in the dorsolateral prefrontal cortex from the CMC identified 12 significant associations, 8 of which also had a significant association in GTEx tissues. Fine-mapping of causal gene sets prioritised gene candidates in 10 Alzheimer’s disease loci with strong evidence for causality. Biological pathway analyses of the meta-analysed GTEx data and CMC data identified a significant enrichment of Alzheimer’s disease association signals in plasma lipoprotein clearance, in addition to multiple immune-related pathways.

**Conclusions:**

Gene expression data from brain and peripheral tissues can improve power to detect regulatory variation underlying Alzheimer’s disease. However, the associations in peripheral tissues may reflect tissue-shared regulatory variation for a gene. Therefore, future functional studies should be performed to validate the biological meaning of these associations and whether they represent new pathogenic tissues.

## Background

An estimated 5.5 million Americans were living with Alzheimer’s disease in 2017, with a prevalence of 10% for people over the age of 65 years [1]. In the absence of a significant medical breakthrough, the number of people living with Alzheimer’s disease is estimated to reach 13.8 million in the USA alone by 2050 [1]. Alzheimer’s disease is the sixth leading cause of death in the USA, but this is likely to be an underestimation as complications of the disease, such as pneumonia, are often recorded as the primary cause of death. Alzheimer’s disease is characterised by neuronal death and key neuropathological changes, including the deposition of β-amyloid and hyperphosphorylated tau tangles. Genome-wide association studies (GWAS) have identified genetic risk factors for Alzheimer’s disease and provided novel insights into disease aetiology. A GWAS meta-analysis of 74,046 individuals (25,580 cases and 48,466 controls) identified 19 genetic risk loci [2], which has since increased to some 24 loci with larger sample sizes [3]. Biological pathway analyses of these data implicate the immune system and lipid metabolism as well as tau binding and amyloid precursor protein metabolism [2], although a disease mechanism of action has yet to be established.

In GWAS, significant associations are reported for a single nucleotide polymorphism (SNP) with the lowest *P* value, but the signal could be explained by one (or more) variant within the linkage disequilibrium block where that SNP resides. Furthermore, GWAS loci may contain multiple genes or regions that affect the expression of other genes. Additional analyses are required to elucidate the biological mechanisms that underlie statistical associations between genetic variants and disease risk. One method is to identify loci where SNP variation is associated with differences in gene expression, called expression quantitative trait loci (eQTL). Genome-wide gene expression data has been successfully integrated with SNP genotype data to prioritise risk genes and reveal possible mechanisms underlying susceptibility to a range of psychiatric disorders [4–7]. This approach may be performed in cases and controls for whom both gene expression and SNP genotype data are available. However, these data sets are likely to have limited sample size and suffer from confounding from reverse causality as variation in gene expression may be influenced by disease status or drug treatment.

An alternative method is to integrate GWAS findings with independent gene expression data provided by large international consortia, such as the multi-tissue Genotype-Tissue Expression (GTEx) project [8] and the CommonMind Consortium (CMC). GTEx (version 7) contains SNP genotype data linked to gene expression across 53 tissues from 714 donors, including 13 brain regions, and the CMC contains gene expression data from the dorsolateral prefrontal cortex of 646 donors. These data represent a valuable resource with which to quantify the association between genetically regulated expression in multiple tissues and the phenotype of interest. Association testing can be carried out using a gene-based approach implemented by transcriptomic imputation approaches [5, 9, 10] which reduce the high level of multiple testing from single-variant tests and increase power to identify trait-associated loci both from a strong functional SNP signal and from a combination of modest signals. The application of transcriptomic imputation using GWAS summary statistics without the need for individual-level data allows these methods to be applied to large-scale GWAS meta-analysis results. Here, we apply a transcriptomic imputation approach called S-PrediXcan to Alzheimer’s disease GWAS summary statistics in order to explore the genetic component of gene expression associated with the disorder. We then use these data in a fine-mapping approach to prioritise candidate causal genes with disease-implicated loci, and identify peripheral tissues that might provide biologically meaningful information on Alzheimer’s disease pathways and processes.

## Materials and methods

### Alzheimer’s disease GWAS summary statistics

Detailed methods, including a description of population cohorts, quality control of raw SNP genotype data, and association analyses for the Alzheimer’s disease GWAS, are described in detail elsewhere [2]. The Alzheimer’s disease GWAS, performed by members of the International Genomics of Alzheimer’s Project (IGAP), included an initial meta-analysis of 4 samples of European ancestry (17,008 cases and 37,154 controls) followed by an analysis of moderately associated SNPs (*P* < 1 × 10^−3^) in an independent sample of 8572 cases and 11,312 controls of European ancestry. All cases received clinical confirmation of late-onset Alzheimer’s disease. SNPs were imputed using the European population reference from the 1000 Genomes Project 2010 interim release based on the sequence data freeze from 4 August 2010 and phased haplotypes from December 2010 [11]. Logistic regression association tests were conducted for imputed marker dosages with age and sex as covariates, as well as principal components to control for possible population stratification. Summary statistics for 7,055,881 autosomal SNPs were made available by IGAP and were utilised in our study.

### Identification of genes with differential expression levels between Alzheimer’s disease cases and controls

We used S-PrediXcan to integrate eQTL information with GWAS summary statistics to identify genes of which genetically predicted expression levels are associated with Alzheimer’s disease status. S-PrediXcan estimates gene expression weights by training a linear prediction model in a reference sample with both gene expression and SNP genotype data. The weights are used to predict gene expression from GWAS summary statistics, while incorporating the variance and co-variance of SNPs from a linkage disequilibrium (LD) reference panel. We used expression weights for 48 tissues with S-PrediXcan expression weights from the GTEx Project (version 7), the dorsolateral prefrontal cortex from the CommonMind Consortium (CMC), and LD information from the 1000 Genomes Project Phase 3 [11]. These data were processed with beta values and standard errors from the Alzheimer’s disease GWAS to estimate the expression-GWAS association statistic. To increase power to identify genes whose expression is similarly differentially regulated across tissues, we meta-analysed the GTEx S-PrediXcan results using the S-MultiXcan algorithm [12]. We used Bonferroni correction to adjust for the number of tests performed within each tissue as well as across all tissues and genes (Table S1).

### Fine-mapping of causal gene sets

S-PrediXcan and other transcriptomic approaches may yield false-positive gene-trait associations due to correlation (LD) among SNPs used to generate the eQTL weights in the predication models [13]. We used fine-mapping of causal gene sets (FOCUS) to appropriately model the impact of gene-trait correlations on the S-PrediXcan expression weights and assign a causal probability to each gene within Alzheimer’s disease risk loci. We used a multi-tissue eQTL reference panel database provided by the authors (https://github.com/bogdanlab/focus/) and LD information from the 1000 Genomes Project Phase 3 [11] as reference genotypes. Chromosome 19 was removed due to the complex association signals within the APOE locus.

### Pathway analysis of gene-based analyses

We performed a biological pathway analysis using generalised linear model regression, with the *Z* score from the GTEx S-MultiXcan or CMC S-PrediXcan association data as the dependent variable and membership in Reactome pathways as a linear predictor. Pathways containing fewer than 10 *cis*-heritable genes (i.e. genes whose average expression across tissues is influenced by proximal [< 1 Mb from the gene start or end] SNPs) were removed, resulting in 1318 biological pathways for pathway enrichment analysis. A Bonferroni-corrected *P* value of *P* = 3.79 × 10^−5^ (adjusting for 1318 tested pathways) was used to correct for multiple testing.

## Results

### A cross-tissue transcriptome-wide association study identifies peripheral tissues enriched with Alzheimer’s disease association signals

**Fig. 1:**
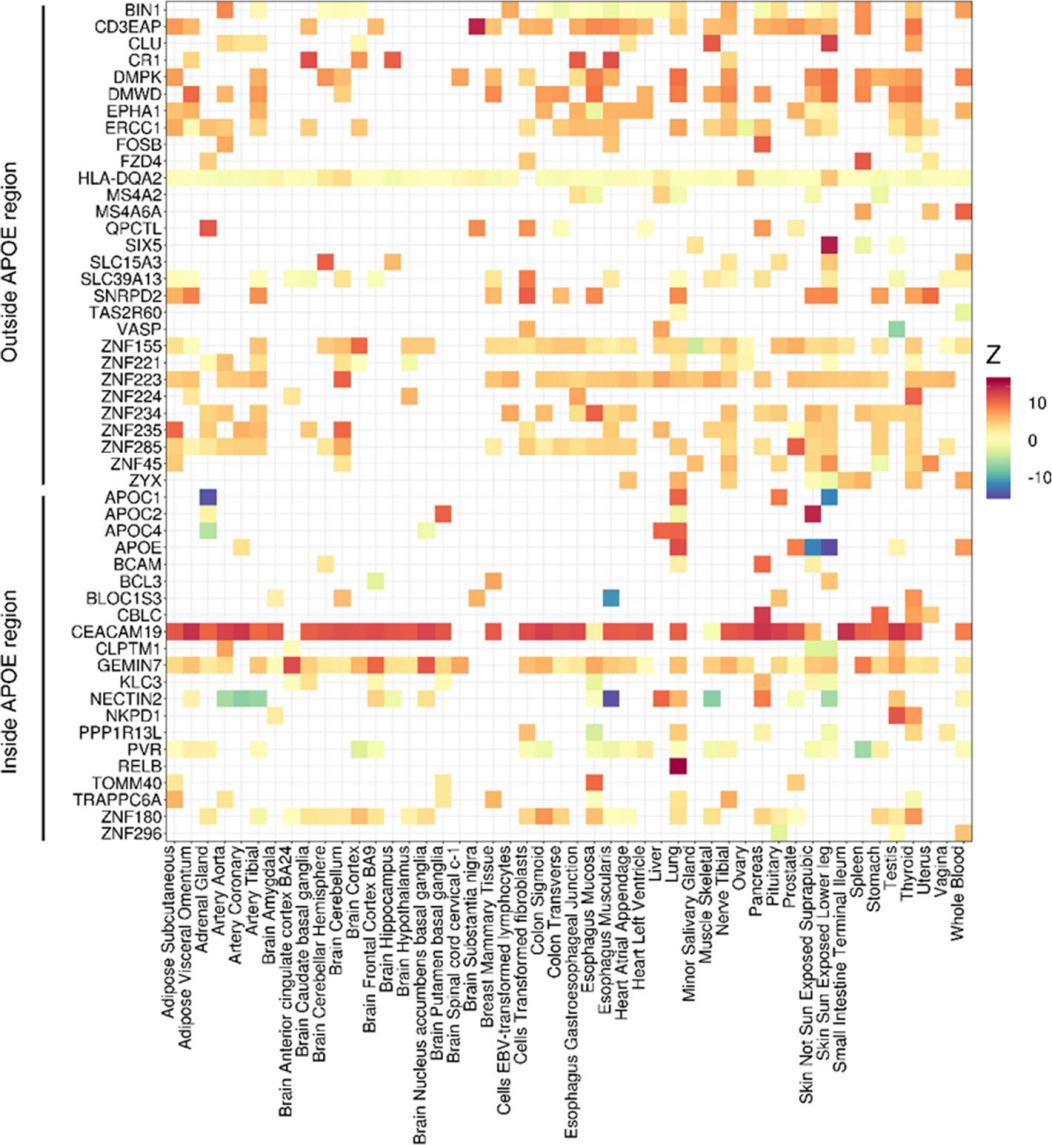
Heatmap of the *Z* score effect directions for significant genes identified in multiple tissues

Using S-PrediXcan, we identified 126 significant associations (Supplementary Table S2) targeting 50 unique genes (Supplementary Table S3) after multiple testing correction for all genes and tissues (*P* < 2.68 × 10^−7^). Among significant associations, there was a slight bias towards positive *Z* scores (*N* = 75 [60%]). The number of significant associations per tissue was largely a function of sample size, with the skin (sun-exposed lower leg) (number of RNA-seq samples *N* = 473) harbouring the largest number of associations (*n* = 9), followed by the lung (*n* = 8) (Table 2) (Supplementary Figure S1). For significant genes identified in multiple GTEx tissues, effect directions were largely consistent across tissues (Fig. 1). The most significant gene association in GTEx data was for *APOE*; genetic variants associated with increased Alzheimer’s disease risk are predicted to downregulate expression levels of *APOE* in three peripheral tissues, including the sun-exposed skin (*Z* = 19.50, *P* = 1.03 × 10^−84^) and the non-sun-exposed skin (− 16.56, *P* = 1.27 × 10^−61^) (Table 1; Supplementary Table S3) after multiple testing correction (Bonferroni correction for 186,230 tests [0.05/186,230] *P* < 2.68 × 10^−7^). Of note, although *APOE* is expressed more widely in the brain compared to most other tissues (Supplementary Figure S2), the eQTL associations with *APOE* are only found in non-brain tissues. While these associations are likely to be due, at least in part, to the increased sample size (and therefore statistical power) of peripheral tissues, they highlight the importance of interrogating multiple (accessible) tissues in eQTL analyses of complex (brain-related) traits.

**Table 1:** Top 5 S-PrediXcan associations by APOE region

Gene	Chr	Most significant tissue	SNPs	Tissues	***Z*** score	***P*** value
**Inside APOE**						
APOE	19	Skin, sun-exposed leg	10	3	− 19.50	1.03 × 10^−84^
NECTIN2	19	Oesophagus muscularis	3	8	− 19.28	8.32 × 10^− 83^
APOC1	19	Adrenal gland	3	3	− 19.13	1.48 × 10^−81^
BLOC1S3	19	Oesophagus muscularis	9	1	− 15.63	4.29 × 10^−55^
RELB	19	Lung	24	1	11.55	7.14 × 10^−31^
**Outside APOE**						
VASP	19	Testis	53	1	− 11.30	1.24 × 10^−29^
SIX5	19	Skin, sun-exposed leg	40	2	10.28	8.60 × 10^−25^
CD3EAP	19	Substantia nigra	6	1	10.06	8.65 × 10^−24^
ZNF155	19	Minor salivary gland	62	2	− 8.45	3.02 × 10^−17^
CLU	2	Skin, sun-exposed leg	5	2	8.22	2.04 × 10^−16^

*Chr* chromosome; *N* SNPs are the number of eQTLs included in the MetaXcan prediction model; *N* tissues are the *N* tissues with *P* value < 7.63 × 10^−7^; *Z* score represents the strength of association between gene expression and disease risk. Positive values indicate that an increased level of gene expression is associated with increased disease risk, while negative values indicate that a reduced level of gene expression increases disease risk

We removed genes flanking the *APOE* region (± 500 kb) due to its strong association with Alzheimer’s disease and identified 29 significant associations (Supplementary Table S3). The most significant gene outside the *APOE* region was the vasodilator-stimulated phosphoprotein *VASP* (*Z* = − 11.30, *P* = 1.24 × 10^−24^) in the testis (Table 1). The most significant association outside chromosome 19 was observed for the clusterin *CLU* in the skin (sun-exposed lower leg) (Table 1; Supplementary Table S3). Taken together with findings for *APOE*, these data suggest the skin (together with other peripheral tissues) may be used as an accessible surrogate tissue for peripheral biomarker discovery and molecular studies of causal disease processes (Table 2).

**Table 2:** Number of significant S-PrediXcan associations per tissue

Tissue	Tissue sample size (*N*)	Gene associations (*N*)	Genes
Skin, sun-exposed lower leg	414	10	APOE, APOC1, NECTIN2, SIX5, CLU, CLPTM1, ZNF229, ZYX, PPP1R13L, KLC3
Lung	383	8	RELB, APOE, CEACAM19, APOC2, APOC1, APOC4, MS4A2, DMPK
Oesophagus mucosa	358	8	PPP1R13L, KLC3, EPHA1, ZNF234, MS4A2, RP11-385F7.1, TOMM40, PVR
Oesophagus muscularis	335	6	NECTIN2, BLOC1S3, CR1, CEACAM19, BIN1, PVR
Skin, not sun-exposed suprapubic	335	6	APOE, APOC2, ZNF229, CLPTM1, MS4A2, PVR
Adrenal gland	175	4	APOC1, APOC4, QPCTL, CEACAM19
Brain hippocampus	111	4	CEACAM19, CR1, NECTIN2, HLA-DQA2
Pancreas	220	4	CEACAM19, CBLC, FOSB, BCAM
Spleen	146	4	PVR, FZD4, CEACAM19, SIX5
Stomach	237	4	MS4A2, ZNF45, CBLC, CEACAM19

To improve power relative to the single-tissue analyses, we combined results from different single-tissue models into a single aggregate statistic using S-MultiXcan. We identified 73 gene-level S-MultiXcan associations after correction for multiple testing (Table 3, Supplementary Table S4), of which 36 were located outside the *APOE* region. The S-MultiXcan analysis identified 27 additional significant genes not found in the single-tissue analyses, 19 of which encoded genes outside the *APOE* region (Supplementary Table S4). The most significant S-MultiXcan association was for *PVRL2* (also known as *NECTIN2*), located within the *APOE* region (oesophagus muscularis; *Z*_mean_ = − 4.94, *P* = 2.64 × 10^−131^), followed by *APOE* (skin, sun-exposed lower leg; *Z*_mean_ = − 3.58, *P* = 4.25 × 10^−101^). The most significant protein coding gene outside the *APOE* region was for protein tyrosine phosphatase, receptor type H *PTPRH* (brain caudate basal ganglia; *Z*_mean_ = 0.35, *P* = 2.19 × 10^−12^). A total of 7 genes were significant in the single-tissue analyses but not in the S-MultiXcan meta-analysis, due in part to heterogeneity in the effect directions of imputed gene expression across tissues.

**Table 3:** Top 5 S-MultiXcan associations by APOE region

Gene	Top tissue	***N*** tissues	***P***	Z score			
				Min	Max	Mean	SD
**Inside APOE**							
PVRL2	Oesophagus muscularis	17	2.64 × 10^−131^	− 19.28	5.78	− 4.94	6.75
APOE	Skin, sun-exposed leg	7	4.25 × 10^−101^	− 19.50	7.51	− 3.58	10.50
APOC1	Adrenal gland	4	4.05 × 10^−92^	− 19.13	5.98	− 6.24	13.43
BLOC1S3	Oesophagus muscularis	6	9.00 × 10^−75^	−15.63	3.48	− 1.78	7.07
APOC4	Adrenal gland	4	1.40 × 10^−39^	− 9.53	5.91	− 0.92	7.96
**Outside APOE**							
SIX5	Skin, sun-exposed leg	4	1.24 × 10^−37^	− 6.18	10.3	− 0.41	7.40
VASP	Testis	3	5.58 × 10^−28^	− 11.3	2.61	− 2.29	7.82
BIN1	Oesophagus muscularis	23	3.58 × 10^−16^	− 6.32	4.09	− 2.21	3.26
CLU	Skin, sun-exposed leg	8	7.51 × 10^−14^	− 3.07	8.22	1.28	4.12
CR1	Oesophagus muscularis	7	1.69 × 10^−11^	− 0.38	7.33	4.299	3.45

*N tissues*, number of tissues with significant gene-based association; *Z score*, minimum, maximum, mean, and standard deviation of the Alzheimer’s disease association coefficient from S-MultiXcan

### A comparison of multi-tissue GTEx results with brain-specific eQTL database from the CommonMind consortium

We performed an S-PrediXcan analysis using expression weights for a single brain region (dorsolateral prefrontal cortex) collected by the CMC and identified 12 significant (*P* < 5.08 × 10^−6^) gene-based associations (Supplementary Table S5). We compared these data with the meta-analysed results from 48 tissues in GTEx (Table 4). Of 12 significant gene-based associations in CMC, 8 also showed a significant association in GTEx tissues (*P* < 1.93 × 10^−6^). The *Z* scores between CMC and GTEx were concordant where the mean absolute GTEx *Z* score was ≥ 1, highlighting the consistency of the datasets. The CMC results were influenced by APOE, with the top association (TOMM40; *P* = 1.37 × 10^−101^) located within the APOE gene cluster on chromosome 19q13. The GTEx tissue with the strongest association for TOMM40 was the oesophagus mucosa (*Z* score = 5.57, *P* = 2.61 × 10^−8^) (Supplementary Table S2), a tissue that contains over twice the number of samples as the largest brain tissue (*N* = 407 versus *N* = 173 in the cerebellum). One TOMM40 association was observed in GTEx brain tissue (putamen basal ganglia); the association was insignificant (*P* = 6.73 × 10^−2^), but the *Z* score direction of effect was consistent with CMC data (GTEx: *Z* = − 1.83; CMC − 21.40).

**Table 4:** Significant associations in the CMC dorsolateral prefrontal cortex and corresponding GTEx association statistics

CMC (DLFPC)			GTEx (48 tissues)			
Gene	***Z***	***P***	Tissue	***Z***	***Z*** SD	***P***
TOMM40	− 21.40	1.37 × 10^−101^	Oesophagus mucosa	0.57	3.44	1.10 × 10^−7^
ZNF222	13.93	4.11 × 10^−44^	–	–	–	–
IRF2BP1	12.28	1.21 × 10^−34^	Heart atrial appendage	− 0.94	0.73	4.34 × 10^−1^
EML2	7.98	1.50 × 10^−15^	Cerebellar hemisphere	− 0.22	2.72	4.01 × 10^−10^
CR1	7.91	2.63 × 10^−15^	Oesophagus muscularis	4.30	3.45	1.69 × 10^−11^
CLPTM1	− 6.30	2.94 × 10^−10^	Skin, sun-exposed leg	− 2.98	4.89	3.35 × 10^−23^
TRAPC6A	− 6.29	3.22 × 10^−10^	Thyroid	− 1.02	2.84	2.26 × 10^−13^
ZNF45	− 6.12	9.18 × 10^−10^	Stomach	0.19	2.72	2.47 × 10^−23^
DMWD	5.91	3.48 × 10^−9^	Adipose visceral omentum	3.45	1.45	3.08 × 10^−8^
ZNF223	5.90	3.69 × 10^−9^	Brain cerebellum	1.03	1.17	5.88 × 10^−4^
PVR	− 4.82	1.41 × 10^−6^	Spleen	− 4.64	2.05	9.68 × 10^−27^
AP2A2	− 4.65	3.30 × 10^−6^	Heart atrial appendage	− 0.30	1.93	2.48 × 10^−2^

*DLPFC* dorsolateral prefrontal cortex

### Fine-mapping further prioritises genes within GWAS risk loci

**Fig. 2:**
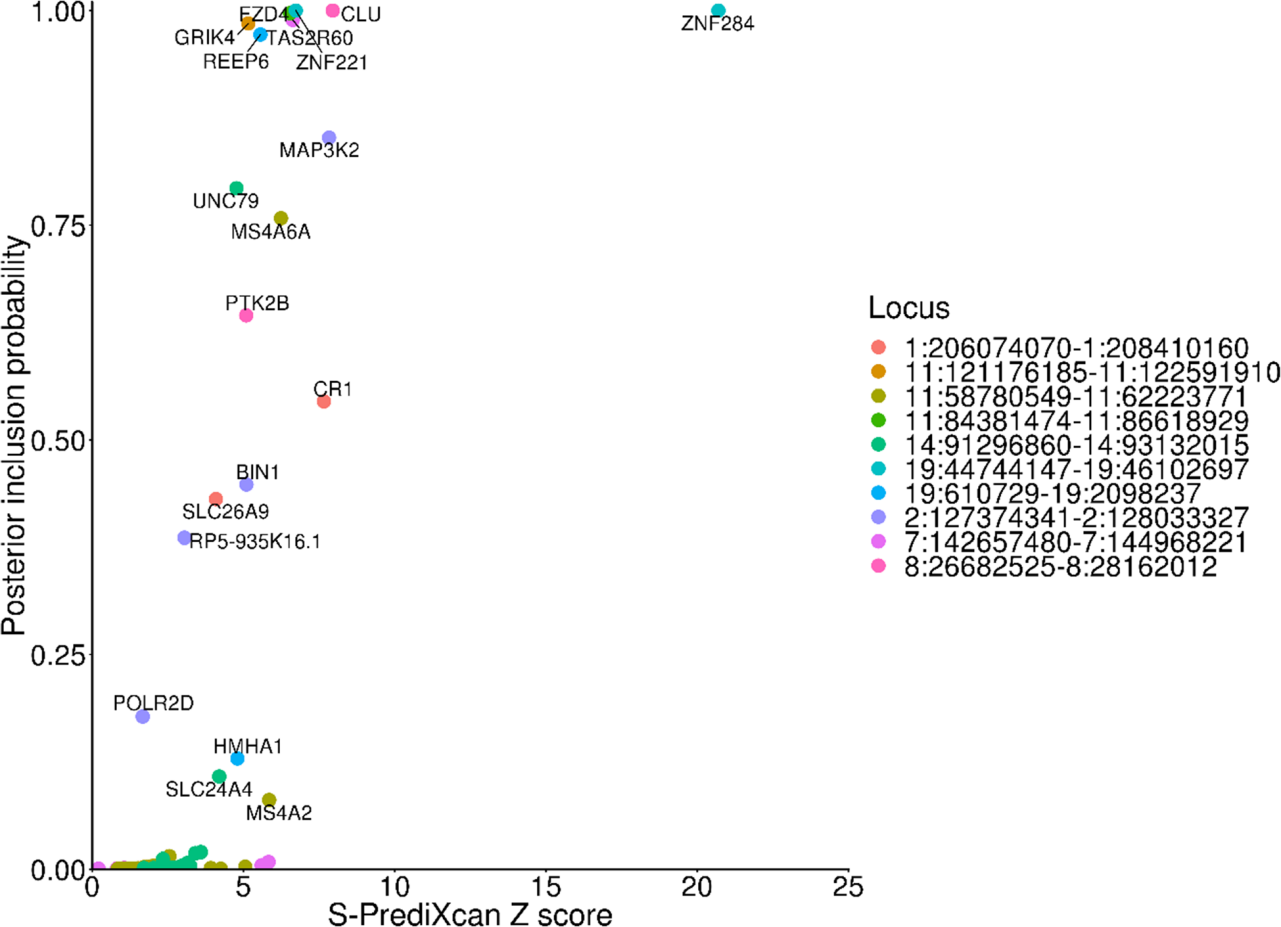
Marginal posterior inclusion probability of credible casual genes versus the S-PrediXcan Z score by chromosomal region

We applied the fine-mapping of causal gene sets (FOCUS) algorithm to prioritise genes within GWAS risk loci. Genes with a higher posterior inclusion probability tended to have a higher S-PrediXcan Z score (Spearman correlation = 0.8269, *P* = 1.64 × 10^− 87^) (Fig. 2). Candidate casual genes not nearest the GWAS index SNP included *GRIK4* (*SROL1* locus; S-PrediXcan Z score − 5.16; PIP 0.985) and *UNC79* (*SLC24A4* locus: S-PrediXcan *Z* score 4.77; PIP 0.793) (Supplementary Table S6). Both *GRIK1* and *UNC79* are involved in ion transmembrane transport and were not prioritised as likely causal genes in a recent GWAS of Alzheimer’s disease [3], highlighting the potential utility of FOCUS in gene prioritisation.

### A biological pathway analysis identifies altered expression of lipoprotein clearance pathways in Alzheimer’s disease

We tested for the enrichment of Alzheimer’s disease associations in Reactome biological pathways by regressing gene pathway membership against the (signed) *Z* score from the S-MultiXcan analyses. This approach allowed us to assess the enrichment of Alzheimer’s disease associations within biological pathways, as well as the mean effect size and effect direction of gene expression within the enriched pathways. In the (multi-tissue) GTEx S-MultiXcan analysis, one pathway—plasma lipoprotein clearance—was significantly downregulated in Alzheimer’s disease cases after correction for multiple testing (beta coefficient = − 0.7861, *P* = 6.64 × 10^−6^) (Table 5, Supplementary Table S7). Plasma lipoprotein clearance was also significantly downregulated in cases using the CMC data (beta = − 0.5646, *P* = 8.31 × 10^−26^). Furthermore, we identified the upregulation of multiple immune-related pathways, especially related to toll-like receptor (TLR) cascades (e.g. toll-like receptor TLR1:TLR2 cascade; beta = 0.3684, 1.32 × 10^−44^) (Table 5, Supplementary Table S8), using the CMC data.

**Table 5:** Biological pathways associated with Alzheimer’s disease association signals in the dorsolateral prefrontal cortex from the CMC

Pathway ID	Pathway name	Coef	SE	*P*
GTEx S-MultiXcan				
R-HSA-8964043	Plasma lipoprotein clearance	− 0.7861	0.1745	6.64 × 10^−6^
CMC DLPFC				
R-HSA-168179	Toll-like receptor TLR1:TLR2 cascade	0.3684	0.0263	1.32 × 10^−44^
R-HSA-167044	Signalling to RAS	0.6713	0.0493	2.78 × 10^−42^
R-HSA-187687	Signalling to ERKs	0.5786	0.0438	6.52 × 10^−40^
R-HSA-447115	Interleukin-12 family signalling	0.6109	0.0468	7.27 × 10^−39^
R-HSA-354192	Integrin alphaIIb beta3 signalling	0.6337	0.0486	8.14 × 10^−39^

*Coef* beta coefficient from a logistic regression model testing the enrichment of genes associated with Alzheimer’s disease in Reactome pathways

## Discussion

We performed multi-tissue analysis of gene expression underlying Alzheimer’s disease to identify and prioritise candidate causal genes and pathogenic tissues. Using the transcriptome-wide association study method S-PrediXcan and tissue-specific eQTL information from GTEx, we identified 50 unique candidate risk genes for Alzheimer’s disease. A meta-analysis of these tissue-specific data found 73 genes associated with Alzheimer’s disease. Because GTEx-derived brain tissues may lack sufficient power to identify robust association signals underlying complex diseases, we ran S-PrediXcan using expression weights derived from 646 dorsolateral prefrontal cortex samples from the CommonMind Consortium. We identified 12 gene-based associations, 8 of which were also significant in the meta-analysed GTEx analysis. Fine-mapping of causal gene sets further prioritised novel gene candidates within 10 independent risk loci. Biological pathway analysis of the meta-analysed GTEx data and CMC data found a downregulation of genes involved in plasma lipoprotein clearance. Furthermore, the CMC data strongly implicated the upregulation of genes involved in immune-related pathways and processes, particularly toll-like receptor activity. These results highlight the utility of investigating multiple tissues underlying complex disorders, including peripheral tissues unrelated to the pathogenic tissue of interest (such as skin tissue for brain-related processes in Alzheimer’s disease) [7]. Our results demonstrate a multi-tissue approach to gene discovery in Alzheimer’s disease may identify not only candidate causal genes and pathways, but peripheral (i.e. accessible) surrogate tissues for diagnostic biomarkers and the discovery of causal mechanisms.

Two recent studies performed transcriptome-wide association analyses of brain samples in Alzheimer’s disease. Raj et al. [14] used TWAS FUSION [15] with eQTL data derived from 450 frontal cortex samples and genotype data from the Religious Order Study or the Memory and Aging Project (ROS/MAP), while Marioni et al. [16] applied Summary-data-based Mendelian Randomization (SMR) [17] to GWAS summary data from a meta-analysis of proxy Alzheimer’s disease cases from the UK Biobank and IGAP meta-analysis summary data, and eQTL data from over 600 frontal cortex samples from the Common Mind Consortium. These analyses identified a total of 9 candidate genes whose expression in brain tissue was associated with Alzheimer’s disease. We found a significant association with 4 of these candidate genes (*CR1*, *TOMM40*, *PVR*, *CLPTM1*) in at least one peripheral tissue. The effect direction of the beta coefficients in our study had the same effect directions for the candidate genes *CR1*, *PVR*, and *CLPTM1*, and the strongest associations were found in peripheral tissues, including the skin.

We observed largely concordant effect directions in the S-PrediXcan association statistics (*Z* scores) across the brain and peripheral tissues. While the brain is the critical pathogenic tissue in Alzheimer’s disease, genetic (i.e. regulatory) effects on gene expression underlying the disorder are enriched in less biologically obvious tissues, such as vascular tissues and the skin [7]. More generally, there is a high level of tissue-shared eQTL regulation at GWAS loci for complex diseases [7], particularly between (embryonically related) brain and skin tissue [18]. These results suggest the study of accessible peripheral tissues such as the skin may capture regulatory effects on gene expression and/or new pathogenic tissues underlying Alzheimer’s disease. Such an approach is supported by surrogate tissue analyses of Parkinson’s disease, which identified alpha-synuclein deposits—a hallmark of dementia—in the nerve fibres of the skin [19, 20]. Future studies of peripheral tissues may therefore increase power to identify gene-based associations in Alzheimer’s disease; however, functional studies will be required to assess the biological relevance on the associations in relation to disease onset and progression.

Transcriptome imputation methods such as S-PrediXcan are prone to false-positive associations due to linkage disequilibrium between SNPs used to build the expression weights, which induce spurious gene-trait associations within chromosomal regions [13]. We used fine-mapping of causal gene sets to further prioritise genes within risk loci. We found the probability for each gene in a region to be causal was largely a function of its S-PrediXcan Z score, where genes with larger *Z* scores had larger posterior inclusion probabilities as the causal gene. Nonetheless, we identified 6 genes that were not reported as the closest gene within ± 100 kb of the top SNP of known GWAS-defined associated genes at the time of publication of Lambert et al. [2], which represent novel, functionally relevant candidate causal genes in Alzheimer’s disease. Among these novel candidates are *GRIK4* at the *SORL1* locus and *UNC79* at the *SLC24A4*-*RIN3* locus. Both *GRIK4* (glutamate ionotropic receptor kainate type subunit 4) and *UNC79* (unc-79 homologue, NALCN channel complex subunit) have biased expression in the brain and encode ion channel subunits, and it is conceivable that their dysfunction may contribute to altered synaptic plasticity, learning, and development in Alzheimer’s disease [21].

Biological pathway analysis of genes in both our meta-analysed GTEx and CMC results found a downregulation of ‘plasma lipoprotein clearance’ in Alzheimer’s disease. These results are consistent with a recent meta-analysis of cross-tissue expression imputation of 44 GTEx tissues [22], which found the enrichment genes whose expression was associated with Alzheimer’s disease in gene ontology terms related to lipoprotein clearance. Lipoprotein clearance may play an important role in Alzheimer’s disease pathogenesis through the association of *APOE* and several other genes that function in lipid or lipoprotein metabolism, including Clusterin (*CLU*) and ATP binding cassette (ABC) transporter A7 (*ABCA7*, [23]). Specifically, it has been hypothesised that dysfunctional lipoprotein clearance in the central nervous system may facilitate the formation of two critical neuroanatomical features in Alzheimer’s disease: amyloid plaques and neurofibrillary tangles. These neuroanatomical features may be indicated by global changes in gene (mRNA) and protein expression of lipid and lipoprotein-related genes in both brain tissue and peripheral blood [24]. The association of lipoprotein-related genes with Alzheimer’s disease in the skin and other non-brain tissues, together with concordant effect directions across tissues (including brain tissue), suggests peripheral tissues may provide a biologically valid substrate for the study of genetic factors and their impact on higher order molecular processes in Alzheimer’s disease.

Pathway analysis of the CMC gene-based data found the upregulation of genes involved in immune-related processes, most notably toll-like receptor cascades. Toll-like receptors are involved in many physiological and pathological responses, and their activity is thought to play a role in several neurological disorders, including Alzheimer’s disease [25, 26]. The receptors are widely expressed on microglial cells—the chief immune cells of the central nervous system—and their activation is associated with Aβ plaque deposition [27] and enhanced neurodegeneration [28]. Although we cannot draw mechanistic conclusions, our results suggest a potential relationship between altered immune signalling, impaired plasma lipoprotein clearance, and Aβ plaque deposition in Alzheimer’s disease.

Our multi-tissue transcriptome imputation approach has a number of advantages over traditional expression quantitative locus studies of complex diseases. First, transcriptome imputation methods allow the study of genetically regulated gene expression without directly measuring expression data from an appropriate cell type in diseased cases and health controls. Second, by estimating the genetically regulated component of gene expression, transcriptome imputation methods remove the impact of unmeasured (i.e. uncontrolled) environmental factors on gene expression, thereby improving the interpretability of expression association signals. Third, transcriptome imputation aggregates SNP-level associations to individual genes, reducing the multiple testing burden and increasing statistical power. A multi-tissue meta-analysis, such as S-MultiXcan, further reduces the multiple testing burden by combining association statistics across all interrogated tissues. Fourth, TWAS methods utilise eQTL information from large eQTL databases with uniform sample collection and strict quality control protocols which improves the reliability of results and enables replication across disorders/traits.

A disadvantage of GTEx is the use of bulk tissue samples to measure gene expression. As a result, GTEx cannot accurately account for cellular heterogeneity and may under represent certain cell populations in a given tissue. Many of the Alzheimer’s disease risk loci identified through GWAS are not highly expressed in bulk brain tissues and therefore previous attempts to identify brain eQTLs have likely been affected by cellular heterogeneity [29, 30]. A large proportion of Alzheimer’s disease risk loci have been linked to immune function, and our results in (dorsolateral prefrontal cortex) brain tissue corroborate these findings. However, the study of immune function in the brain is complicated by the heterogeneous cell populations, which may dilute disease-specific expression signatures from immune-related populations such as microglia. Analyses of primary cell-type-specific expression from the Immune Variation project have shown that Alzheimer’s disease risk alleles are enriched among monocyte-specific eQTLs [31]. Future studies may therefore use more easily accessible monocytes as a proxy to examine the (immune) cell-specific effects of susceptibility variants in Alzheimer’s disease.

## Conclusions

In summary, we performed a multi-tissue transcriptome-wide association study of Alzheimer’s disease. We confirmed an association between DNA sequence variation and gene expression for known Alzheimer’s disease candidate genes and identified multiple genes whose expression has not previously been associated with the disease. Many disease associations were observed in both brain and peripheral tissues, most notably skin tissue, and the effect directions for the association statistics were largely consistent across tissues. A meta-analysis of 48 GTEx tissues, including 13 brain tissues, confirmed the association of candidate genes identified in the single-tissue analyses, in addition to several novel genes, most of which were also identified in an analysis of gene expression in the dorsolateral prefrontal cortex from the CMC. These results suggest the joint analysis of brain and peripheral tissues may capture regulatory effects underlying Alzheimer’s disease; however, functional studies will be required to assess their biological relevance in disease activity. The use of skin tissue—which had the largest number of associations with Alzheimer’s disease in our study—represents a promising avenue for the study of regulatory variation and risk genes in Alzheimer’s disease.

## Supplementary information

**Additional file 1: Supplementary Table 1.** Bonferroni corrected *P* value thresholds after adjusting for the number of genes expressed in a given tissue (i.e. 0.05/number of genes in tissue).

**Additional file 2: Supplementary Table 2.** Single-tissue S-PrediXcan results across 48 GTEx tissues.

**Additional file 3: Supplementary Table 3.** Single-tissue S-PrediXcan results across 48 GTEx collapsed by gene.

**Additional file 4: Supplementary Table 4.** Significant S-TissueXcan results.

**Additional file 5: Supplementary Table 5.** Single-tissue S-PrediXcan results from dorsolateral prefrontal cortex (CMC).

**Additional file 6: Supplementary Table 6.** Fine-mapping of causal gene sets (FOCUS) of Alzheimer’s disease GWAS summary statistics.

**Additional file 7: Supplementary Table 7.** Pathway analysis of GTEx S-TissueXcan results.

**Additional file 8: Supplementary Table 8.** Pathway analysis of CMC S-PrediXcan results.

**Additional file 9: Supplementary Table 9.** Pathway analysis of GTEx S-TissueXcan results, no APOE region.

**Additional file 10: Supplementary Table 10.** Pathway analysis of CMC S-PrediXcan results, no APOE region.

**Additional file 11: Supplementary Figure 1.** Number of significant S-PrediXcan associations against GTEx tissue sample size.

**Additional file 12: Supplementary Figure 2.** Expression of APOE (ENSG00000130203.5) across GTEx tissues.

## Data Availability

All data generated or analysed during this study are included in this published article and its supplementary information files.
