# Supplementary figures and images for: An analysis of genetically regulated gene expression across multiple tissues implicates novel gene candidates in Alzheimer’s disease
Source: Alzheimers Res Ther. 2020 Apr 16;12:43. doi: 10.1186/s13195-020-00611-8 (PMC7164172; doi:10.1186/s13195-020-00611-8)

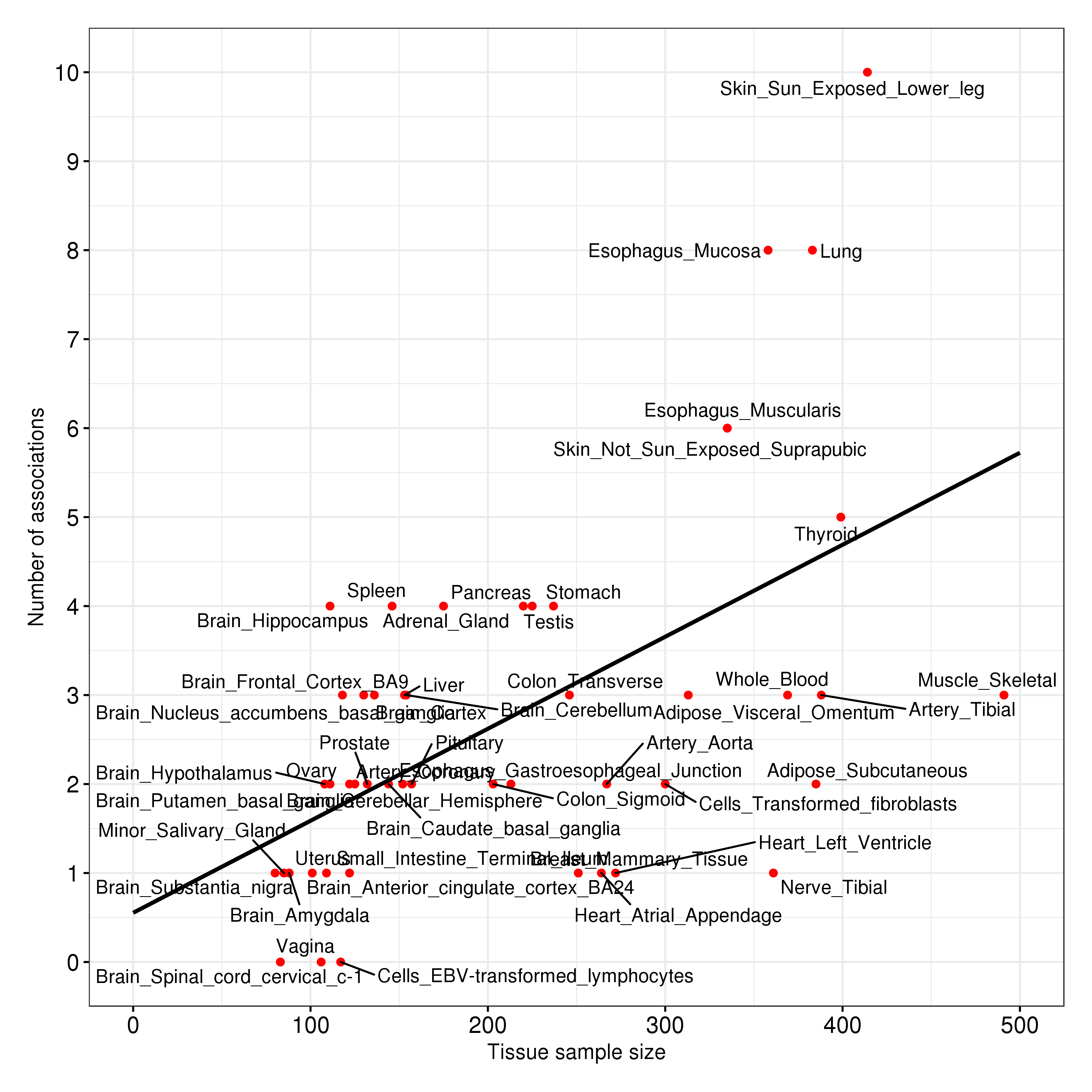

Supplement: Supplementary file 11 — Additional file 11: Supplementary Figure 1. Number of significant S-PrediXcan associations against GTEx tissue sample size. [file 13195_2020_611_MOESM11_ESM.tiff]

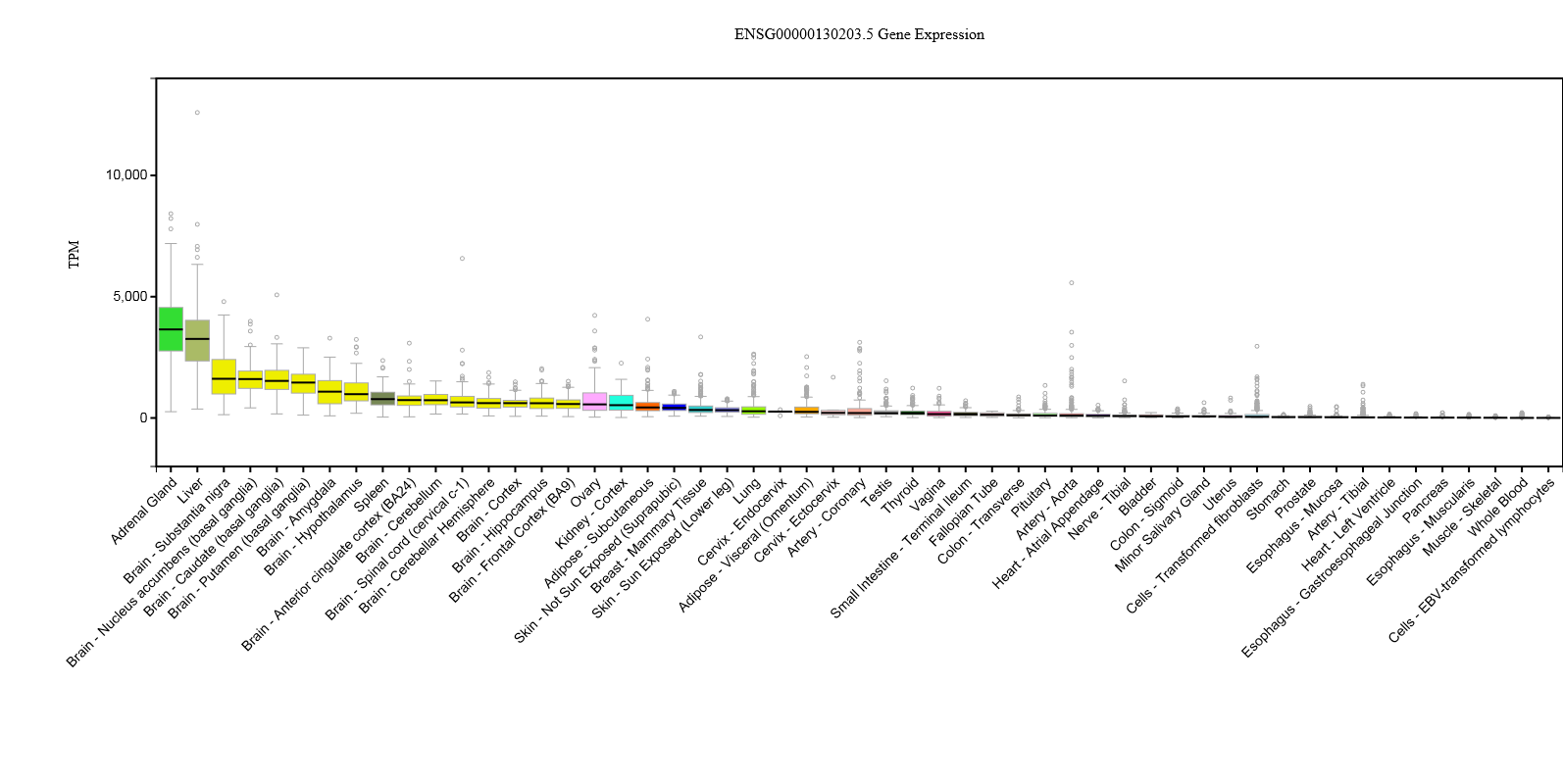

Supplement: Supplementary file 12 — Additional file 12: Supplementary Figure 2. Expression of APOE (ENSG00000130203.5) across GTEx tissues. [file 13195_2020_611_MOESM12_ESM.png]
